# Evaluation of Risk Factors Associated with Endometriosis in Infertile Women

**DOI:** 10.22074/ijfs.2016.4763

**Published:** 2016-04-05

**Authors:** Mahnaz Ashrafi, Shahideh Jahanian Sadatmahalleh, Mohammad Reza Akhoond, Mehrak Talebi

**Affiliations:** 1 Department of Endocrinology and Female Infertility, Reproductive Biomedicine Research Center, Royan Institute for Reproductive Biomedicine, ACECR, Tehran, Iran; 2 Department of Obstetrics and Gynecology, Faculty of Medicine, Iran University of Medical Science, Tehran, Iran; 3 Department of Reproductive Health and Midwifery, Faculty of Medical Sciences, Tarbiat Modares University, Tehran, Iran; 4 Department of Statistics, Mathematical Science and Computer Faculty, Shahid Chamran University, Ahwaz, Iran

**Keywords:** Case-Comparison Study, Endometriosis, Infertility, Symptoms

## Abstract

**Background:**

Endometriosis affects women’s physical and mental wellbeing. Symptoms include dyspareunia, dysmenorrhea, pelvic pain, and infertility. The purpose of this study is to assess the correlation between some relevant factors and symptoms and risk of an endometriosis diagnosis in infertile women.

**Materials and Methods:**

A retrospective study of 1282 surgical patients in an infertility Institute, Iran between 2011 and 2013 were evaluated by laparoscopy. Of these, there were 341 infertile women with endometriosis (cases) and 332 infertile women with a normal pelvis (comparison group). Chi-square and t tests were used to compare these two groups. Logistic regression was done to build a prediction model for an endometriosis diagnosis.

**Results:**

Gravidity [odds ratio (OR): 0.8, confidence interval (CI): 0.6-0.9, P=0.01], parity (OR: 0.7, CI: 0.6-0.9, P=0.01), family history of endometriosis (OR: 4.9, CI: 2.1-11.3, P<0.001), history of galactorrhea (OR: 2.3, CI: 1.5-3.5, P=0.01), history of pelvic surgery (OR: 1.9, CI: 1.3-2.7, P<0.001), and shorter menstrual cycle length (OR: 0.9, CI: 0.9-0.9, P=0.04) were associated with endometriosis. Duration of natural menstruation and age of menarche were not correlated with subsequent risk of endometriosis (P>0.05). Fatigue, diarrhea, constipation, dysmenorrhea, dyspareunia, pelvic pain and premenstrual spotting were more significant among late-stage endometriosis patients than in those with early-stage endometriosis and more prevalent among patients with endometriosis than that of the comparison group. In the logistic regression model, gravidity, family history of endometriosis, history of galactorrhea, history of pelvic surgery, dysmenorrhoea, pelvic pain, dysparaunia, premenstrual spotting, fatigue, and diarrhea were significantly associated with endometriosis. However, the number of pregnancies was negatively related to endometriosis.

**Conclusion:**

Endometriosis is a considerable public health issue because it affects many women and is associated with the significant morbidity. In this study, we built a prediction model which can be used to predict the risk of endometriosis in infertile women.

## Introduction

Endometriosis is the benign proliferation of functioning endometrial glands and stroma, located outside of the uterine cavity. It is diagnosed by laparoscopic observation of lesions, nodules, or cysts on the pelvic peritoneum or the pelvic organs (1), and is one of the most common diseases in gynecology field (2), as well as a source of an exorbitant economic burden in public health field (3). Endometriosis could be considered as an epigenetic, hormonal regulated disease (4,5) which is progesterone resistance, and estrogens promote perilesional angiogenesis and neo-innervation and allow endometriotic foci to growth. Moreover, estrogens may contribute to decreases in the local immune surveillance by Peritoneal Fluid Mononuclear Cells (6,7) and enhance the pro-inflammatory microenvironment typical of the disease (8,9). Endometriosis, as an enigmatic disease, is responsible for chronic pelvic pain, dysmenorrhea, menorrhagia, dyspareunia and infertility (2,10,12). The range of the variable influence on the resulting pain syndrome in endometriosis is very wide, for example, classified according to the revised American Society for Reproductive Medicine (rASRM) classification, previous surgical procedures, Douglas obliteration, extent of the sub-peritoneal infiltration and pelvic wall implants (13). It has been observed that there is no relation between the intensity of the pain experienced and stages of disease. Regardless of disease stage, women with endometriosis seem to have similar menstrual patterns and ages at menarche (12). Some studies have revealed an increased risk of other diseases among the women with endometriosis (14,15). Approximately, one half of the infertile women facing surgery are diagnosed with endometriosis (16). Despite this relatively high prevalence and morbidity, little information has been published about the risk factors for endometriosis in infertile women, who are more likely to have endometriosis as an underlying cause of their infertility. 

The relationship between endometriosis in infertile women and clinical symptoms is a complex association, which is influenced by multiple factors including psychological, different cultural conditions, ethnic, and climatic conditions. Therefore, the aim of this study is to determine the demographic, personal characteristic, reproductive variables, contraception and menstruation pattern associated with the presence of endometriosis in infertile women. We also investigated the parameters that might predict the risk of an endometriosis diagnosis. 

## Materials and Methods

In retrospective study, the subjects were 1282 currently infertile [the failure to achieve a clinical pregnancy after 1 year or more of regular unprotected sexual intercourse (17) women aged 16-46 years who underwent laparoscopy between 2011 and 2013. The study was approved by the Institutional Review Board of the Royan Institute Research Center and the Royan Ethics Committee according to the Helsinki Declaration; signed informed written consent was obtained from all participants. The information was collected by using a self-administered questionnaire, which included questions about demographic characteristics, family history, health of reproductive and infertility, symptoms and physical characteristics. Characteristics of the menstrual cycle included age at menarche, average length of menstrual bleeding and cycle, previous use of oral contraceptives (OC, total number of years taken), previous usage of intrauterine device (IUD), age at first pregnancy and pelvic pain. 

**Fig 1:**
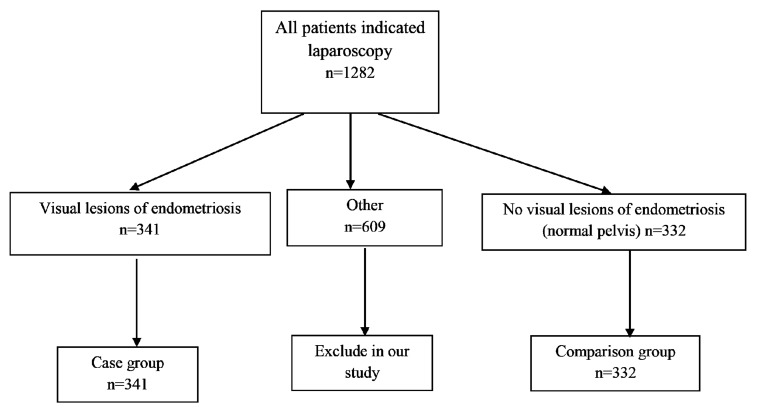
Flow chart showing longitudinal analysis of the population.

The most common indications for laparoscopy were as follows: symptoms of endometriosis, such as dyspareunia, dysmenorrhea and pelvic pain, unexplained factor in infertility, uterine abnormality and tubo-peritoneal disorder. After laparoscopy, we divided the 1282 participants into three groups, shown in the diagram. The first group of 341 patients had visual lesions of endometriosis (52 had stage I, 85 had stage II, 111 had stage III, and 84 had stage IV); the second group of 609 patients had adhesions, fibroids, leiomyomas, and/or uterine abnormalities; the third group of 332 subjects had no visual lesions of endometriosis, i.e., a normal pelvis without any complications (Fig .1). In analysis, the second group was excluded in order to evaluate the risk factors in women with endometriosis (pure endometriosis) compared with the group with a normal pelvis and no other complications. 

All clinicians of the study were required to collect the following information from all participants: history of infertility, gravidity, parity, ectopic pregnancies, abortion, pathologies of the reproductive tract ( e.g., sexually transmitted diseases, pelvic inflammatory disease (PID), and salpingitis), any observations during surgery (e.g., uterine anomalies, tubal obstruction, leiomyoma, and adhesions), and any medication taken on a regular basis. Weight and height of all female infertility referring to the Center are measured during the first visit. Body mass index (BMI) is defined as weight divided by height squared (kg/m^2^)

Symptoms of endometriosis (in last 6 months) were collected, such as dysmenorrhoea, pelvic pain, dyspareunia, premenstrual spotting, fatigue, diarrhea and constipation. Other symptoms indicative of endometriosis are as follows: irregular bleeding, severe bleeding, bloat, nausea, vomiting, dyschezia and dysuria. 

Following surgery, the stage of the disease was defined according to the rASRM as stage I (minimal), stage II (mild), stage III (moderate), and stage IV (severe) (18). In 80.2% of women with endometriosis, histologic confirmation was also made. 

Statistical analysis was performed by using SPSS program (Version 18, Chicago, IL, USA), comparing those with a diagnosis of endometriosis and those with a normal pelvis using Chisquare or t tests, as appropriate. In order to predict a diagnosis of endometriosis, we used logistic regression. The data were expressed as means ± SD. Odds ratio (OR) and 95% confidence interval (95% CIs) were also calculated for each factor. In order to build a prediction model, we used stepwise logistic regression in a backward manner. In this model, a P value of 0.15 was used as entry criterion, whereas a P value of 0.1 was the threshold for a variable to stay in the model. The area under the receiver operating characteristic (ROC) curve (AUC) shows the discriminative performance of the fitted logistic model. An AUC equal to 0.5 shows no discriminative performance, whereas an AUC of 1.0 indicates perfect discrimination. In ordinal regression analysis, predictors which probably reflect symptoms of endometriosis (such as dyspareunia, dysmenorrhea and pelvic pain) were examined. P values less than 0.05 were considered to be statistically significant. Moreover, we assessed the calibration of the model by comparing the predicted probability in a category of patients and the observed percentage of endometriosis in that category. According, we categorized the predicted probabilities in 10 groups, based on percentile points with steps of 10% per step. In each category, we compared the mean predicted probability in that particular category with the observed probability, i.e., the number of women with endometriosis in that category divided by the total number of women in that category. The results were plotted graphically. 

## Results

The prevalence of endometriosis in the total sample of women undergoing laparoscopy was 26.5%. The women’s demographic and reproductive characteristics are listed in Table 1. The study group (cases) consisted of 341 infertile women, who were diagnosed to have endometriosis by laparoscopy. The severity of the disease was staged according to the rASRM classification of endometriosis. Endometriosis was staged as 15.2% minimal (rASRM stage I), 24.9% mild (rASRM stage II), 32.5% moderate (rASRM stage III) and 24.3% severe (rASRM stage IV). All these women were infertile [primary infertility in 296 (89.2% of women) and secondary infertility in 36 (10.8% of women)], with mean age of 32.4 ± 4.9 years, mean age at menarche of 13.1 ± 1.2 years, mean duration of infertility of 5.8 ± 1.6 years (Table 1).

As a comparison, the 332 infertile women who referred to the same center for infertility and were laparoscopically confirmed to be without endometriosis were included. All these women were infertile [primary infertility in 296 (87.4%) of women and secondary infertility in 34 (12.6%) of women], with mean age of 31.4 ± 5.2 years, mean age at menarche of 13.1 ± 1.3 years and mean duration of infertility of 6.0 ± 1.8 years (Table 1).

Independent t test analysis showed no significant difference in BMI between the case and the comparison groups (P>0.05). Those with endometriosis did not differ from the comparison group with regard to age at menarche, menstrual status, duration of menstrual bleeding, type of infertility, duration of infertility and cigarette smoking. In contrast, a significant difference was found concerning the length of menstrual cycles, age, gravidity and history of abortion (Table 1).

We also found no association between endometriosis and the use of IUD or previous exposure to OCs. No significant difference was found between the two groups in previous cervical trauma, genital tract abnormality, history of PID and sexually transmitted diseases (STD) (i.e., chlamydia, herpes, condylomas, and gonorrhea).

**Table 1:** Selected demographic, personal, and lifestyle characteristics of the case and the comparison groups


Parameters	Camparison group	Cases	OR(95%CI)	P value

Age (Y)				
<30	155(45.5)	118(35.5)	1†	
30-35	103 (30.2)	126 (38)	1.6 (1.1- 2.2)	0.02
>30	83(24.3)	88(26.5)	1.3(0.9-2.04)	
Age at menarche (Y)	13.1 ± 1.3	13.1 ± 1.2	0.9 (0.8-1.10)	0.7
Age at marriage (Y)	20.3 ± 3.8	22.1 ± 4.6	1.1 (0.9-1.2)	0.8
Night worker				
No	338(99.1)	325(97.9)	1†	
Yes	3(0.9)	7(2.1)	2.4(0.6-9.4)	0.2
Menstruation status				
Irregular	47 (86.2)	42 (12.7)	1†	0.6
Regular	294 (13.8)	290 (87.3)	1.10 (0.7-1.7)	
Menstrual cycle length	31.1 ± 7.8	29.9 ± 7.5	0.9 (0.9-0.9)	0.04
Duration of bleeding menstrual (days)	6.0 ± 1.8	5.8 ± 1.6	0.9 (0.8-1.05)	0.4
History of live birth				
Yes	43 (12.6)	36 (10.8)	1†	
No	298 (87.4)	296 (89.2)	1.1 (0.7-1.9)	0.4
Type of infertility				
Secondary	43 (12.6)	36 (10.8)	1†	0.4
Primary	298 (87.4)	296 (89.2)	1.1 (0.7-1.9)	
Duration of infertility (Y)	6.90 ± 4.3	6.7 ± 4.5	0.9 (0.9-1.03)	0.8
Contraceptive				
None	189 (55.4)	167 (50.3)	1†	
OCP	26 (7.6)	27 (8.1)	1.1(0.6-2.09)	0.2
IUD	8 (2.3)	3 (0.9)	0.4 (0.1-1.6)	
Other	118 (34.6)	135 (40.7)	1.2 (0.9-1.7)	
Duration of consume contraceptive (month)	30.9 ± 20.09	30.2 ± 20.1	0.9(0.9-1.009)	0.7
Gravidity	0.5 ± 1.1	0.3 ± 0.8	0.8 (0.6-0.9)	0.01
Parity	0.4 ± 1.08	0.3 ± 0.6	0.7 (0.6-0.9)	0.01
BMI (kg/m2)	25.6 ± 3.8	25.05 ± 4.01	0.9 (0.9-1.002)	0.06
No. of spontaneous abortion	0.4 ± 1.06	0.2 ± 0.5	0.7 (0.5-0.8)	0.02
Smoking				
No	337 (98.8)	326 (98.2)	1†	
Yes	4 (1.2)	6 (1.8)	1.5 (0.4-5.5)	0.5
Family history of endometriosis				
NO	337(97.4)	301(90.7)	1†	<0.001
Yes	7(2.1)	31(9.3)	4.9(2.1-11.3)	
History of galactoreahea				
No	302(88.6)	255(76.8)	1†	<0.001
Yes	39(11.4)	77(23.2)	2.3(1.5-3.5)	
Abnormality genital tract				
No	259(76)	272(81.9)	1†	0.05
Yes	82(24)	60(18.1)	0.06(0.4-1.01)	
History of STD				
No	340 (99.7)	331 (99.7)	1†	0.9
Yes	1 (0.3)	1 (0.3)	1.02 (0.06-16.4)	
History of PID				
No	337(99.8)	325(97.9)	1†	0.3
Yes	4(1.2)	7(2.1)	1.8(0.5-6.2)	
History pelvic surgery				
No. surgery	126(37)	77(23.2)	1†	
Laparascopy	180(52.8)	189(56.9)	1.7(1.2-2.4)	
Laparoscopy and laparotomy	11 (3.2)	42 (12.7)	1.6 (0.8-3.08)	<0.001
Laparotomy	24 (7)	24 (7.2)	6.2 (3.03-12.8)	
Previous cervical trauma				
No. trauma	332(97.7)	317(95.5)	1†	0.1
Trauma	9(2.6)	15(4.5)	1.7(0.7-4.04)	


Data are presented as n (%) or mean ± SD. †; Reference category, OR; Odds ratio, CI; Confidence interval, OCP; Oral contraceptives, IUD; Intrauterine device, BMI; Body mass index, STD; Sexually transmitted disease and PID; Pelvic inflammatory disease.

However, patients with endometriosis were significantly more likely to have a family history of endometriosis, a history of galactorrhea, and a history of pelvic surgery (Table 1).

Symptom distribution among patients with early-stage (stage I or II disease) and late-stage (stage III or IV disease) endometriosis is summarized in Table 2, which shows that dysmenorrhea, dyspareunia, pelvic pain, premenstrual spotting, fatigue, diarrhea and constipation were more common among late-stage endometriosis patients than in those with early-stage endometriosis and more prevalent among patients with endometriosis than those with a normal pelvis (Table 2).

Finally, in order to build a prediction model and to find the most important factors affecting endometriosis, we used a logistics regression model in a backward manner. Table 3 shows the result of fitting logistic regression model to the data.

In the logistic regression model, gravidity, family history of endometriosis, history of galactorrhea, history of pelvic surgery, dysmenorrhoea, pelvic pain, dysparaunia, premenstrual spotting, fatigue, and diarrhea were significantly positively associated with endometriosis. However, number of pregnancies was negatively related to endometriosis (Table 3).

**Table 2:** Distribution of symptoms associated with endometriosis according to disease stage and comparison group


Parameters	Disease stage				Comparison grou[	P value	OR (95%CI)

Dysmenorrhoea							
Yes	28(53.8)	52(61.2)	79(71.2)	68(81)	140(41.1)		3.1(2.2-4.2)
No	24(46.2)	33(38.8)	32(28.8)	16(19)	201(58.9)	<0.001	1†
Dysparunia							
Yes	15(28.8)	32(37.6)	39(35.1)	46(54.8)	68(19.9)		2.6(1.8-3.7)
No	37(71.2)	53(62.4)	72(64.9)	38(45.2)	273(80.1)	<0.001	1†
Pelvic Pain							
Yes	16(30.8)	56(34.1)	52(46.8)	44(52.4)	68(19.1)		3.1(2.2-4.4)
No	36(69.2)	29(65.9)	59(53.2)	40(47.6)	276(80.9)	0.02	1†
Premenstrual spotting							
Yes	7(13.5)	27(31.8)	31(27.9)	41(48.8)	42(12.3)		3.3(2.2-4.6)
No	45(86.5)	58(68.2)	80(72.1)	43(51.2)	299(87.7)	<0.001	1†
Fatigue							
Yes	3(5.8)	12(14.1)	12(10.8)	20(23.8)	16(4.7)		3.3(1.8-6.0)
No	49(94.2)	73(85.9)	99(89.2)	64(76.2)	325(95.3)	<0.001	1†
Diarrhea							
Yes	2(3.8)	6(7.11)	8(7.2)	9(10.7)	1(0.3)		27.6(3.7-205.5)
No	50(96.2)	79(92.9)	103(92.8)	75(89.3)	340(99.7)	<0.001	1†
Constipation							
Yes	3(5.8)	16(22.4)	13(11.7)	16(19)	32(9.4)		1.7(1.09-2.8)
No	49(94.2)	66(77.6)	98(88.3)	68(81)	309(90.6)	0.003	1†


†; Reference category, OR; Odds ratio and CI; Confidence interval.

**Fig 2:**
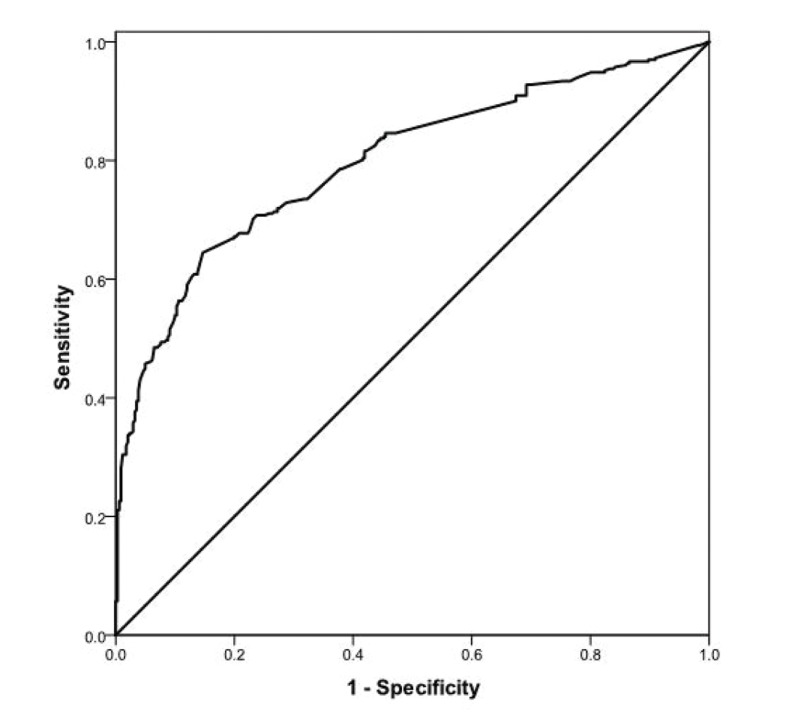
Receiver operating characteristic (ROC) curve for assessment discriminative preformance of logistic regression.

The AUC shows the discriminative performance of the logistic model. The AUC value equal to 0.5 shows no discriminative performance, while AUC value of 1.0 indicates perfect discrimination. The AUC value for the fitted logistic model was 0.78 (95% CI 0.74-0.81), showing a good predictive performance for the fitted logistic regression model (Fig .2). We checked the goodness of fit of our model by the Hosmer- Lemeshow goodness of fit test. The P value for this test was 0.13 showing good predictive performance of our model. Also validity of the model was assessed by calibration plot; all predicted probabilities were almost similar to the observed rate of endometriosis in each group and show the good calibration of the model.

**Table 3:** Result of fitting multiple logistic regression


Parameters	OR (95% CI)	P value

Gravidity	0.8 (0.6-0.9)	0.04
Family history of endometriosis	2.7 (1.06-7.1)	0.03
History of galactoreahea	1.8 (1.1-3.05)	0.01
History of pelvic surgery		
No. surgery	1†	<0.001
Laparoscopy	3.4 (1.5-7.8)	
Laparotomy	4.1 (2.4-6.8)	
Laparoscopy and laparotomy	14.5 (6.1-34.2)	
Dysmenorrhoea	1.8 (1.1-2.8)	0.006
Pelvic pain	4.1 (2.4-6.8)	<0.001
Dysparunia	1.6 (1.09-2.4)	0.01
Premenstrual spotting	2.2 (1.3-3.6)	0.002
Fatigue	2.6 (1.3-5.1)	0.006
Diarrhea	19.06 (2.4-150.6)	0.005
Area under ROC curve (95% CI)=0.78 (0.74-0.81)		


†; Reference category, OR; Odds ratio, CI; Confidence interval and ROC; Receiver operating characteristic.

## Discussion

Endometriosis is one of the most common gynecological diseases in the different countries. It is a confusing disease with little known about its distribution, true prevalence and risk factors in the population (19). In our study, prevalence of endometriosis in the total sample of women undergoing laparoscopy was 26.5%. Ozkan et al. (20) found endometriosis has a prevalence of 25-40% in infertile women. The goal of this study was to investigate the relation between some relevant factors and risk of an endometriosis diagnosis. Our study represents an analysis of 673 infertile women undergoing laparoscopy (cases and comparison group) for further understanding of the different risk factors and associated symptoms of endometriosis with infertility. 

A significant difference was observed between the average age of infertile women with and without endometriosis (32.4 ± 4.9 vs. 31.4 ± 5.2). Increasing age, alcohol use, low body weight, family history of endometriosis, early menarche, prolonged menstrual flow, and short cycle interval, intercourse during menses, infertility are also alleged risk factors (21,22). Our results show no significant difference association between BMI or smoking intake and endometriosis. This is consistent with the several studies showing no association with these parameters and endometriosis (22,23). Some authors have reported inverse relation between BMI and endometriosis (24,25), although in these latter studies the comparison group was not infertile women. 

The present study indicated a higher rate of endometriosis among more educated women (data not shown), is consistent with the results of other studies (2,26). The possible association of endometriosis with higher education level is due to a delay in childbearing. The association between education level and endometriosis probably reflects the socioeconomic issues, such as access to the medical care. The absence of gravidity in endometriosis group was associated with significantly increased odds of suffering from endometriosis; a finding that is consistent with several other reports (26,27). However, spontaneous abortions and ectopic pregnancy were not linked to endometriosis (14). In the present study, we did not find any significant difference between duration of infertility and endometriosis. Akande et al. (28) reported that the effects of duration of infertility and primary infertility were not observed to be statistically significant for women with endometriosis. Several studies show that prolonged duration of infertility itself may be a precursor of endometriosis in the absence of other causes (29,30). Duration and heaviness of flow and premenstrual spotting were also risk factors for endometriosis (P<0.05). The majority of studies to date have reported that early menarche (<11 years) increases the risk of endometriosis (23,31,32), but our result did not find any significant difference between age of menarche and endometriosis. Peterson et al. (33) reported that there was no relationship between endometriosis and menstrual cycle history, including average cycle length, number of menstrual cycles, and age at menarche. 

Cramer et al. (32) found in their case-control study that women with infertility associated with endometriosis had a lower age at menarche, shorter menstrual cycles and longer duration of menstrual bleeding than those of the control group. Likewise, we found no significant difference between the heavy menstrual flow and the risk of endometriosis. Treloar et al. (34) have also reported the same result, even though heavy flow is associated with endometriosis in the other studies (1,35). A recent study has reported that a shorter cycle length is associated with an increasing risk of endometriosis, but none of these studies has examined specifically this association before the onset of symptoms (1,36). Overall, these findings support the Sampson’s theory of retrograde menstruation, in which women with greater opportunity for menstrual contamination of the pelvis are at increased risk of endometriosis (34,37). 

We found significant correlation between length of cycle and the presence of endometriosis, but other studies reported no significant association between length of cycle, length of menses, as well as age at menarche with the presence of endometriosis (2,34). Multiple lines of evidence have indicated that endometriosis is associated with increased exposure to menstruation, an assumption supporting the retrograde menstruation theory (1,36). Menstrual factors, previously shown to be associated with endometriosis, include early menarche, shorter cycle length, longer menses, late pregnancies and longtime delay between first pregnancy and menarche (2). However, the association between menstrual factors and endometriosis remains unclear because some studies fail to show a relationship between these factors and the disease (27,38). Nevertheless, other studies found that women with endometriosis reported apparently either shorter or longer and heavier menstruation than normal cycle (39,40). Stovall et al. (41) have found menstrual cycle ≤27 days as a risk factor that seems to be associated to endometriosis in infertile women. 

The results of the present study showed that the stage of endometriosis, according to the rASRM classification, is related to the presence of pain. We found a significant difference between stage and symptoms of endometriosis. 

Vercellini et al. (42) reported that endometriosis associated symptoms of endometriosis, such as dyspareunia, dysmenorrhea and pelvic pain and endometriosis stage is directly related to the persistence of that symptom. Arruda et al. (43) reported disease stage was significantly associated with severity of dysmenorrhoea and nonmenstrual pain. 

The Italian study (44) showed no relationship between the intensity of pain and the stage of the disease. In another study, on 469 women aged 1845 years old revealed that no clear-cut association between stage, site or morphological characteristics of pelvic endometriosis and pain (45). 

In our study, infertile women who experienced dysmenorrhea were more likely to have endometriosis rather than women reported no pain during their menstruation. Therefore, if more severe dysmenorrhea is associated with increased risk of contractility and expulsion menstrual debris into the pelvic, severe cramps may suggest susceptibility to the disease (1). Pelvic pain is often used as a diagnostic tool for endometriosis with dysmenorrhea being the most commonly reported symptom (14,27). Pelvic pain might predispose women to endometriosis via retrograde menstruation (23). There was also a significant difference of increasing risk of endometriosis with the reported pelvic pain, dysmenorrheal and dyspareunia (12). In our study, we found significant difference between endometriosis and pelvic pain occurring during ovulation in contrast to what was found by Treloar et al. (34). The cul-de-sac and uterosacral ligaments are the most common sites of endometriosis (46). Dyspareunia may be common complaint among women with endometriosis because these areas are stretched during intercourse (9). 

Use of contraception, as OCs and IUD, are also known to affect menstrual flow. If retrograde menstruation is involved in the etiology of endometriosis, usage of IUD (a common cause of menorrhagia) would be expected to increase the risk of the disease. Hughes et al. (47) have suggested that use of IUD not influence the development of endometriosis. In other study, OC exposure was associated with a lower risk of endometriosis (48). Our results indicate no significant difference between IUD and OCs exposure and endometriosis in infertile women. 

In the present study, we observed a positive correlation between the previously operated pelvic and endometriosis. In addition, family history of endometriosis was prevalent among the patients with the disease compared with patients with a normal pelvis (P<0.001). This point was also confirmed by the other authors (49,50). These data appear to confirm that a familial tendency toward endometriosis, and also suggest that genetic risk factor in the pathogenesis of endometriosis exist (33). 

Interestingly, in our study, the endometriosis group commonly reported constipation (61.4 vs. 38.6%) and diarrhea (96.2 vs. 3.8%), suggesting that irritable bowel syndrome be considered as a co-morbidity.

Finally, various factors may be useful in screening for endometriosis and predict risk of an endometriosis diagnosis. Patient characteristics, gravidity, family history of endometriosis, history of galactorrhea, history of pelvic surgery, dysmenorrhoea, pelvic pain, dysparaunia, premenstrual spotting, fatigue, diarrhea, and the number of pregnancies have been evaluated as predictors of endometriosis. Thus, infertility center should have enough information about the symptoms of endometriosis in order to provide more information for patients. The findings suggest that gravidity, family history of endometriosis, history of galactorrhea, history of pelvic surgery, dysmenorrhoea, pelvic pain, dysparaunia, premenstrual spotting, fatigue, and diarrhea were significantly positively associated with endometriosis. However, number of pregna ncies was negatively related to endometriosis.

Our models can be used with the almost and highest reliability as a guide to screen for endometriosis, in patients comparable to the developing population. The effects of using these models in patient care have to be further investigated. In addition to high prevalence of endometriosis, consultation in relation to risk factors for endometriosis will be helpful for early detection and prevention of disease.

Only a prospective cohort study can specify to what extent any of these characteristics indicate risk-factors for different stages of endometriosis. Although our results show that there is a relationship between pain symptoms, disease severity and infertility, it may also help to focus the future of epidemiologic studies regarding prevention and treatment for the endometriosis.

## Conclusion

There is a decreasing risk of endometriosis in currently infertile women with history of pregnancy and an increased risk in infertile women reporting a history of dysmenorrhea, family history of endometriosis, history of galactorrahea, history of pelvic surgery, dysmenorrhoea, pelvic pain, dysparunia, premenstrual spotting, fatigue and diarrhea. Due to the high prevalence of endometriosis, consultation in relation to risk factors for endometriosis, we will be helpful for early screening, detection and prevention of disease.
